# The Crystallization Behavior of a Na_2_O-GeO_2_-P_2_O_5_ Glass System: A (Micro)Structural, Electrical, and Dielectric Study

**DOI:** 10.3390/ma17020306

**Published:** 2024-01-07

**Authors:** Sara Marijan, Marta Razum, Kristina Sklepić Kerhač, Petr Mošner, Ladislav Koudelka, Jana Pisk, Andrea Moguš-Milanković, Željko Skoko, Luka Pavić

**Affiliations:** 1 Division of Materials Chemistry, Ruđer Bošković Institute, Bijenička 54, 10000 Zagreb, Croatia; smarijan@irb.hr (S.M.); mrazum@irb.hr (M.R.); ksklepickerhac@gmail.com (K.S.K.); mogus@irb.hr (A.M.-M.); 2 Department of General and Inorganic Chemistry, Faculty of Chemical Technology, University of Pardubice, 53210 Pardubice, Czech Republic; petr.mosner@upce.cz (P.M.); ladislav.koudelka@upce.cz (L.K.); 3 Department of Chemistry, Faculty of Science, University of Zagreb, Horvatovac 102a, 10000 Zagreb, Croatia; jana.pisk@chem.pmf.hr; 4 Department of Physics, Faculty of Science, University of Zagreb, Bijenička 32, 10000 Zagreb, Croatia

**Keywords:** phosphate glasses, phosphate glass-ceramics, controlled crystallization, (micro)structure-property relationship, IR-ATR, PXRD, SEM-EDS, Solid-State Impedance Spectroscopy (SS-IS)

## Abstract

Sodium-phosphate-based glass-ceramics (GCs) are promising materials for a wide range of applications, including solid-state sodium-ion batteries, microelectronic packaging substrates, and humidity sensors. This study investigated the impact of 24 h heat-treatments (HT) at varying temperatures on Na-Ge-P glass, with a focus on (micro)structural, electrical, and dielectric properties of prepared GCs. Various techniques such as powder X-ray diffraction (PXRD), infrared spectroscopy-attenuated total reflection (IR-ATR), and scanning electron microscopy-energy dispersive spectroscopy (SEM-EDS) were employed. With the elevation of HT temperature, crystallinity progressively rose; at 450 °C, the microstructure retained amorphous traits featuring nanometric grains, whereas at 550 °C, HT resulted in fully crystallized structures characterized by square-shaped micron-scale grains of NaPO_3_. The insight into the evaluation of electrical and dielectric properties was provided by Solid-State Impedance Spectroscopy (SS-IS), revealing a strong correlation with the conditions of controlled crystallization and observed (micro)structure. Compared to the initial glass, which showed DC conductivity (*σ*_DC_) on the order of magnitude 10^−7^ Ω^−1^ cm^−1^ at 393 K, the obtained GCs exhibited a lower *σ*_DC_ ranging from 10^−8^ to 10^−10^ Ω^−1^ cm^−1^. With the rise in HT temperature, *σ*_DC_ further decreased due to the crystallization of the NaPO_3_ phase, depleting the glass matrix of mobile Na^+^ ions. The prepared GCs showed improved dielectric parameters in comparison to the initial glass, with a noticeable increase in dielectric constant values (~20) followed by a decline in dielectric loss (~10^−3^) values as the HT temperatures rise. Particularly, the GC obtained at @450 stood out as the optimal sample, showcasing an elevated dielectric constant and low dielectric loss value, along with moderate ionic conductivity. This research uncovers the intricate relationship between heat-treatment conditions and material properties, emphasizing that controlled crystallization allows for precise modifications to microstructure and phase composition within the remaining glassy phase, ultimately facilitating the fine-tuning of material properties.

## 1. Introduction

The ever-expanding energy demands arising from modern lifestyles and rapid advancements within the electronics sector underscore the need for the development of novel, sustainable, and renewable materials specifically tailored for integration into electronic devices and circuits. This necessity covers a wide range of applications, from solid-state batteries (SSBs) [1] and capacitors [2] to microelectronic packaging substrates [3] and humidity sensors [4,5]. In the pursuit of high-efficiency materials, considerable efforts have been channeled towards exploring inorganic materials, with a notable focus on oxide glasses and glass-ceramics (GC). These materials garner significant interest in electrical technologies due to their dense and uniform microstructures, marked by a low grain boundary effect, along with their inherent capability for precise adjustment to achieve optimal properties, including robust mechanical and thermal stability, as well as exceptional electrical and dielectric properties [6,7,8]. In contrast to oxide glass, where physicochemical properties are primarily determined by chemical composition, GCs offer a unique advantage in a way that their properties are influenced not only by composition or crystallographic structure but also by microstructure and the precise type and quantity of crystalline phase(s) within the residual glassy phase. The latter can be precisely controlled by adjusting heat-treatment (HT) conditions, including temperature and duration.

One property that exhibits a strong dependence on the controlled crystallization process is ionic conductivity, and multiple studies have consistently confirmed a significant enhancement in ionic conductivity in the resulting GC [6]. Amongst the studied systems, NASICON-type phosphates have received particular attention owing to their exceptional conductivity, reaching levels on the order of 10^−3^ S cm^−1^ [9]. Within this group, the LiGe_2_(PO_4_)_3_-based systems have been the subject of extensive investigation [10,11,12,13,14,15,16], showcasing promise as solid electrolyte materials [17,18]. Thorough research into this system has persistently demonstrated that precise control over crystallization leads to a notable enhancement in ionic conductivity, with temperature and HT duration emerging as pivotal factors not only in the formation of conductive crystal phases but also in determining the size of resulting crystal grains and the porosity of the samples [12,13,14,15,16]. Although it has been recognized that increased conductivity is often associated with nanocrystallization [6], a study of the LiAlGePO_4_ system by Cruz et al. [12] revealed that nanometric grain sizes do not necessarily promote electrical conductivity, and the highest conductivity values were achieved in a GC sample with a micrometric grain size and porous microstructure. Besides Li–Ge–P-based GC systems, their sodium counterparts, like the one from the NaSnGePO_4_ system [19], have been gaining growing interest due to their potential use in sodium SSBs, which are increasingly recognized as a safer, cost-effective, and environmentally friendly alternative to Li-based SSBs [20,21]. Beyond their role as electrolyte materials, sodium phosphate compounds have emerged as highly prospective candidates for ionically conducting inorganic binders in electrode materials, further enhancing their potential in battery technology [22]. In addition to its binding role, the binder should possess intrinsic ionic conductivity, which is expected to facilitate ion transport between electrolyte and electrode particles. Moreover, higher dielectric constant values of binding materials are anticipated to promote ion dissociation and separation, thus contributing to the overall improvement in electrode performance [23].

Besides their use in SSBs, glasses-(ceramics) with low dielectric constants are sought-after as substrate materials for high-speed microelectronic packaging [3,24]. Reducing the dielectric constant of these materials typically involves utilizing less polarizable lighter such elements as Na, P, and O, primarily due to the influence of factors such as atom size and electron number density. Another effective approach for achieving a lower dielectric constant involves increasing porosity through controlled crystallization, providing a convenient means to modify the dielectric properties of these materials. On the other hand, porous GCs can also demonstrate favorable humidity-sensing properties, as water molecules have the capacity to physically adsorb on the grain surface within the pores, depending on the relative humidity [25,26]. An example of such a system is the Na–Mo–P GCs from our prior research, where it was demonstrated that the samples with a porous microstructure exhibit outstanding humidity-sensing capabilities [27]. Building upon our research group’s prior work, which demonstrated the efficacy of Solid-State Impedance Spectroscopy (SS-IS) for the comprehensive analysis of electrical and dielectric properties in phosphate-based glasses [28,29] and GC materials [30,31], including its utility in studying dielectric relaxation phenomena, assessing the dielectric loss factor, and distinguishing between bulk and grain boundary contributions, we chose to investigate the electrical and dielectric properties of GCs prepared from the 40Na_2_O-*x*GeO_2_-(60-*x*)P_2_O_5_ glass system described in [28].

This study investigated the impact of HT temperature on the (micro)structural, electrical, and dielectric properties of GCs produced through the controlled crystallization of an initial glass with a nominal composition of 40Na_2_O-10GeO_2_-50P_2_O_5_ whose structural, thermal, and electrical properties are detailed in [28]. The structural and microstructural properties of the prepared GCs were examined through powder X-ray diffraction (PXRD), infrared spectroscopy-attenuated total reflection (IR-ATR), and scanning electron microscopy-energy dispersive spectroscopy (SEM-EDS). The findings reveal that, as the HT temperature rises, there is a gradual increase in crystallinity. Moreover, the microstructure undergoes a transition, evolving from a primarily amorphous glass matrix with nanometric crystalline grain sizes in the NGP@450 GC to a fully crystallized microstructure featuring large grains with micron-scale dimensions in the NGP@550 GC. Solid-State Impedance Spectroscopy (SS-IS) was employed to study the electrical and dielectric properties, and the results underscore their strong correlation with the (micro)structural characteristics. In the crystallized samples, the DC conductivity experienced a decline in comparison to the initial glass. Among the GCs under examination, the NGP@450 stood out with the highest DC conductivity, measuring 2.30 × 10^−8^ Ω^−1^ cm^−1^ at 393 K. On the other hand, with an increase in the HT temperature to 550 °C, a GC sample NGP@550 was produced, which exhibited the lowest values for dielectric parameters, including a dielectric permittivity of 19.37 and a dielectric loss of 0.008. This research elucidates the intricate relationship between HT temperature and material properties, arising from the changes in the microstructure, emphasizing the vast potential of these materials across various technological applications.

## 2. Materials and Methods

Within the glass series 40Na_2_O-*x*GeO_2_-(60-*x*)P_2_O_5_ (*x* = 0–30, mol%) described in [28], a glass with the composition 40Na_2_O-10GeO_2_-50P_2_O_5_ (referred to as the initial glass and denoted in mole fractions of individual oxides) was chosen for the study of the crystallization process and the impact of (micro)structural changes on the electrical and dielectric properties of the prepared glass-ceramics (GCs). The starting glass, labeled as NGP, was prepared from raw materials through the melt quenching technique according to the procedure described in [28]. The conditions for the controlled crystallization of NGP glass were selected based on the results of the differential thermal (DTA) analysis [28], and the heat treatment (HT) was performed for 24 h at temperatures of 450 °C, 500 °C, and 550 °C. As a result, three distinct GCs, named NGP@450, NGP@500, and NGP@550, were synthesized. GCs formed through processing @450 °C and @500 °C exhibited a whitish/brown-whitish color and maintained their structural integrity after thermal treatment. These samples were compact and easily prepared for structural analysis and electrical measurements. However, the GC obtained @550 °C deformed during the HT process, resulting in an exceptionally thin and fragile sample that presents challenges when preparing it for electrical measurements.

The structure of the glass and glass-ceramic samples was examined using IR-ATR spectroscopy with a Thermo Fisher Nicolet iS50 FT-IR instrument (Thermo Fisher Scientific Inc., Waltham, MA USA). The crystalline phases formed during thermal treatment and their weight percentages were determined via powder X-ray diffraction (PXRD) analysis using Bruker D8 Discover diffractometer with a LYNXEYE XE-T detector (Bruker AXS GmbH, Karlsruhe, Germany). PXRD patterns were recorded in the range of 10° ≤ 2*θ* ≤ 70° with CuKα radiation (1.5418 Å). Rietveld structure refinement was conducted using the HighScore X’pert Plus program 3.0 (Malvern Panalytical, Almelo, The Netherlands). Scanning electron microscopy with energy dispersive X-ray spectroscopy (SEM-EDS) analysis was performed with a field-emission scanning electron microscope (FE-SEM) JSM-7000 (JEOL, Welwyn Garden City, UK) to gain detailed insights into the morphology and composition of prepared samples. Gold electrodes were deposited on both sides of the samples using a Sputter Coater SC7620 (Quorum Technologies LTD, Newhaven, UK). The electrical and dielectric properties of both the initial glass and the prepared GCs were examined by Solid-State Impedance Spectroscopy (SS-IS) using an impedance analyzer Novocontrol Alpha—AN dielectric spectrometer (Novocontrol Technologies GmbH & Co., KG, Hundsangen, Germany). Complex impedance, Z*(*ω*), was measured across a wide frequency range (0.01 Hz–1 MHz) and temperature range (273–523 K), and the measurement results were processed and analyzed using WinFIT software (version 3.2, Novocontrol Technologies GmbH & Co. KG, Hundsangen, Germany). Complex electrical conductivity, σ*(*ω*), permittivity, ε*(*ω*), and electrical modulus, M*(*ω*), were calculated from the experimental values of real and imaginary components of complex impedance, along with sample geometry.

## 3. Results and Discussion

### 3.1. Structural and Microstructural Analysis

#### 3.1.1. PXRD Analysis

**Figure 1:**
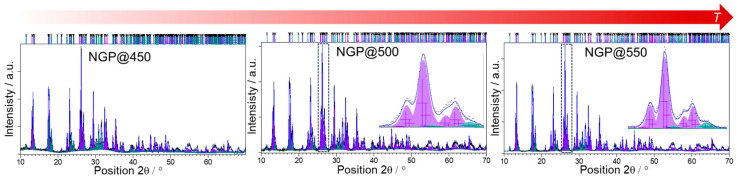
Rietveld refinement of prepared glass-ceramics. Experimental data are represented by black lines, while the calculated profiles are shown in blue. Diffraction lines belonging to the NaPO_3_ phase are colored purple, while GeP_2_O_7_ lines are shown in teal. The insets in NGP@500 and NGP@550 patterns demonstrate the differences in the integral line breadths with the increase in temperature.

The PXRD analysis shows that all three GCs contained identical crystalline phases, namely NaPO_3_ (ICSD-18139) [32] and GeP_2_O_7_ (ICSD-74876) [33], see Figure 1. In contrast to the PXRD pattern of NGP@450, the PXRD patterns of NGP@500 and NGP@550 did not exhibit the typical amorphous halo, usually observed between 20–30°. This absence indicates a minimal, if not negligible, presence of any remaining amorphous phase.

Quantitative phase analysis performed with Rietveld refinement showed NaPO_3_ to be the dominant crystal phase in all three samples, with GeP_2_O_7_ being the minor one. In the NGP@450 sample, the weight fraction of the NaPO_3_ crystal phase was 75.0(8)%, and the GeP_2_O_7_ crystal phase constituted 25.0(9)%. Furthermore, in the NGP@500 sample, the weight fraction of the NaPO_3_ was 76.4(7)%, while GeP_2_O_7_ crystal constituted 23.5(9)%. At the highest temperature, the ratio of the two crystal phases remained similar, with 74.3(8)% of NaPO_3_ and 25.7(9)% of GeP_2_O_7_. R_wp_ factors for the refinements of diffraction data of these three samples were 10.7, 10.9, and 10.0, respectively. These results indicate that elevating the crystallization temperature has a negligible impact on the quantitative composition, i.e., the ratio of crystalline phases. However, it can be observed that the amorphous halo, quite pronounced at 450 °C, diminishes at higher temperatures, while the width of diffraction lines becomes narrower. These observations were further reinforced by the IR-ATR spectroscopy results, as illustrated in Figure S1a in the Supplementary Material (SM). It is noteworthy that the IR-ATR spectroscopic results of the initial NGP glass closely align with prior findings from Raman spectroscopy [28], indicating a predominance of metaphosphate structure with traces of pyrophosphate units [34,35]. In the case of NGP@450, these results revealed a significant presence of the glass matrix, coupled with subtle changes in band shapes that signal the initial stages of crystallization. In contrast, the spectra of NGP@500 and NGP@550 exhibited remarkable similarity, characterized by sharp signals typically associated with crystalline materials, indicating a notably high degree of crystallinity. These findings are in alignment with the PXRD analysis. Moreover, when the IR-ATR spectra of the prepared samples were compared to the spectrum of the NaPO_3_ crystal phase from the SpectraBase^®^, a notable agreement was revealed, providing additional support for the PXRD analysis results that emphasize the prevalence of the NaPO_3_ crystal phase.

#### 3.1.2. SEM-EDS Analysis

**Figure 2:**
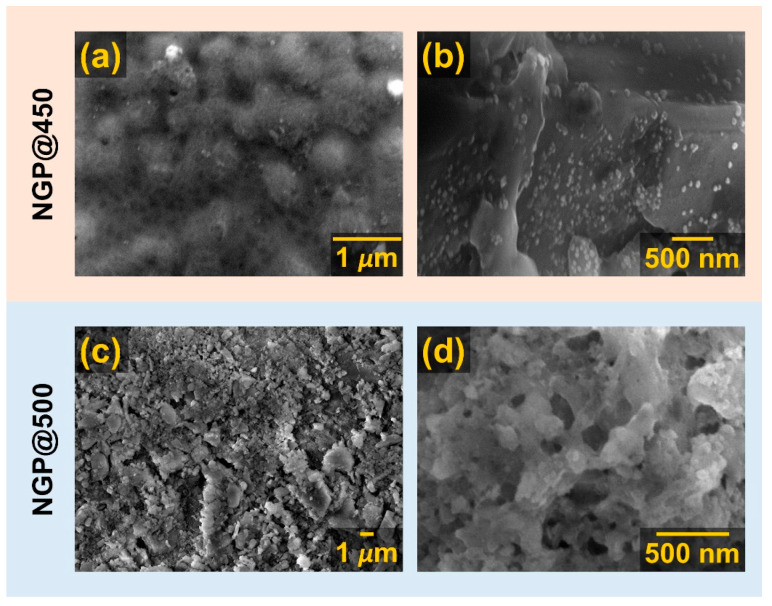
SEM micrographs of the (**a**,**b**) NGP@450 and (**c**,**d**) NGP@500 glass-ceramic surface.

Upon examining the acquired SEM micrographs, it became apparent that the surface of NGP@450 lacked visible crystals when observed at lower magnifications (×5k), see Figure S2a in the SM. Instead, aggregates resembling phase separations within the amorphous matrix were observed at higher magnifications (×25k), see Figure 2a and Figure S2b in the SM. However, at the highest magnifications of ×30k, randomly dispersed nanosized grains were distinguished, measuring less than 50 nm, growing on the predominant amorphous glass matrix’s surface, see Figure 2b and Figure S2c in the SM.

**Figure 3:**
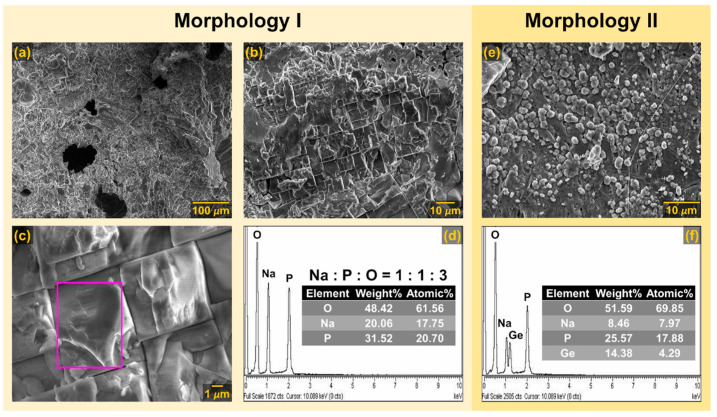
SEM micrographs and EDS spectra of the NGP@550 glass-ceramic surface. Morphology I of regular square-shaped crystalline grains with micron-scale dimensions is depicted in (**a**–**c**), and the EDS spectrum corresponding to the region marked with a purple rectangle in (**c**) is provided in (**d**). Morphology II, depicting smaller spherical grains, is shown in (**e**), with the corresponding EDS spectrum presented in (**f**). The tables in the inset of EDS spectra in (**d**) and (**f**) display the weight and atomic percentages of the detected elements.

The observed grains can be attributed to the prevalent NaPO_3_ crystalline phase, as supported by the PXRD results for NGP@450, see Figure 1. Additionally, the EDS analysis results indicate that the GC obtained through HT at the lowest temperature of 450 °C predominantly consisted of an amorphous matrix, with germanium detected on all analyzed surfaces, see Figure S2 in the SM. Upon raising the crystallization temperature to 500 °C, the surface of NGP@500 exhibited randomly scattered grains with sizes ranging from 100 to 500 nm, lacking a distinct morphology, as shown in Figure 2c,d. The EDS analysis of grains on the surface of NGP@500 revealed an approximate Na:P:O ratio of 1:1:3, see Figure S3 in the SM, providing further validation to the PXRD and IR-ATR analysis results, which indicated the presence of the dominant crystalline phase, NaPO_3_. Unlike the previous two GC samples, the NGP@550 displayed conspicuous surface features: large crystalline grains with micron-scale dimensions, as depicted in Figure 3a–c. These grains had a well-defined shape, predominantly appearing as regular squares (Morphology I), see Figure 3c. In the EDS analysis of this region, Na, P, and O elements, with a Na:P:O ratio of 1:1:3, were exclusively detected, see Figure 3d, thus unequivocally confirming that the square-shaped crystalline grains were indeed of the NaPO_3_ phase, which constituted the majority of the NGP@550, as evidenced by PXRD and IR-ATR. However, a different crystal morphology (Morphology II) in the shape of smaller spherical grains was also evident, as shown in Figure 3e. The results of EDS analysis conducted in this region revealed a significant presence of Ge, alongside Na, P, and O elements, see Figure 3f, suggesting that Morphology II may be associated with the minor GeP_2_O_7_ crystalline phase.

It is noteworthy that the EDS analysis results revealed a similarity in elemental compositions between NGP@500 and NGP@550 samples with the dominant crystalline NaPO_3_ phase. However, while the grains in the NGP@500 lacked a regular shape, the GC obtained at the higher crystallization temperature of 550 °C exhibited a distinct, well-defined square shape. On the other side, while Ge was present on all analyzed surfaces of the NGP@500, it was either absent or found in minimal quantities in the NGP@550. This may be attributed to the reduced presence of a glass matrix or its absence in the latter sample. The SEM-EDS analysis hence clearly demonstrates the progressive increase in crystallinity and the decrease in the amount of the residual amorphous phase in the prepared samples as the HT temperature rises. It is noteworthy that raising the HT temperature from 500 to 550 °C had no discernible impact on the composition and amount of crystalline phases. However, it did significantly influence the microstructure of the resulting GCs, a factor that will be discussed further due to its significant effect on electrical transport.

### 3.2. Electrical Analysis

#### 3.2.1. Electrical Conductivity

**Figure 4:**
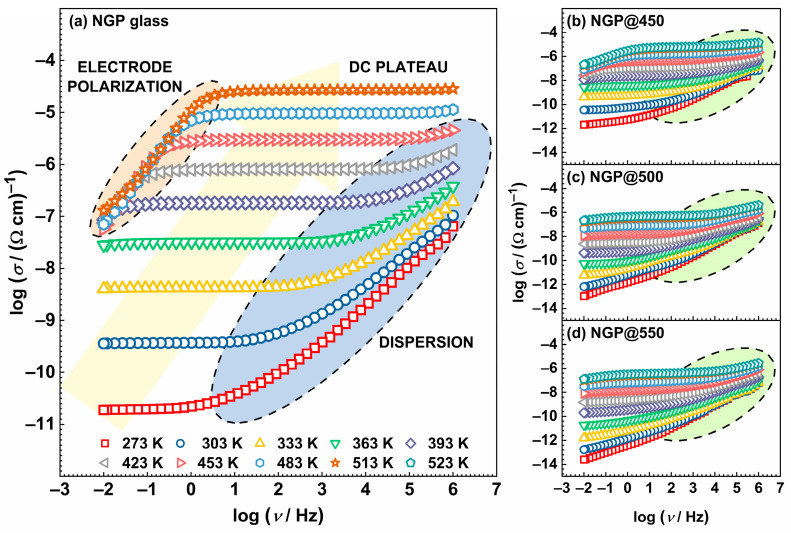
Conductivity spectra of the initial NGP glass (**a**) and prepared glass-ceramics (**b**–**d**).

Figure 4 shows the conductivity spectra of the initial NGP glass and all prepared GC samples. In the presented spectra for the initial glass, see Figure 4a, three different areas were distinguished. At low temperatures and frequencies, conductivity demonstrated a frequency-independent behavior due to long-range ion motion, resulting in a constant value referred to as the DC plateau, *σ*_DC_. Conversely, at low frequencies and higher temperatures, conductivity decreased due to electrode polarization (EP), which is a consequence of Na^+^ ion accumulation on gold electrodes. At high frequencies, a dispersion region emerged, indicating short-range ion motion. The difference in the shape of the spectra of the initial glass, see Figure 4a, and GCs, see Figure 4b–d, was most pronounced in the dispersion part of the curves and was caused by the different contributions (amorphous phase, crystalline grains, grain boundaries, etc.) to the total conductivity.

#### 3.2.2. Complex Impedance Plane

**Figure 5:**
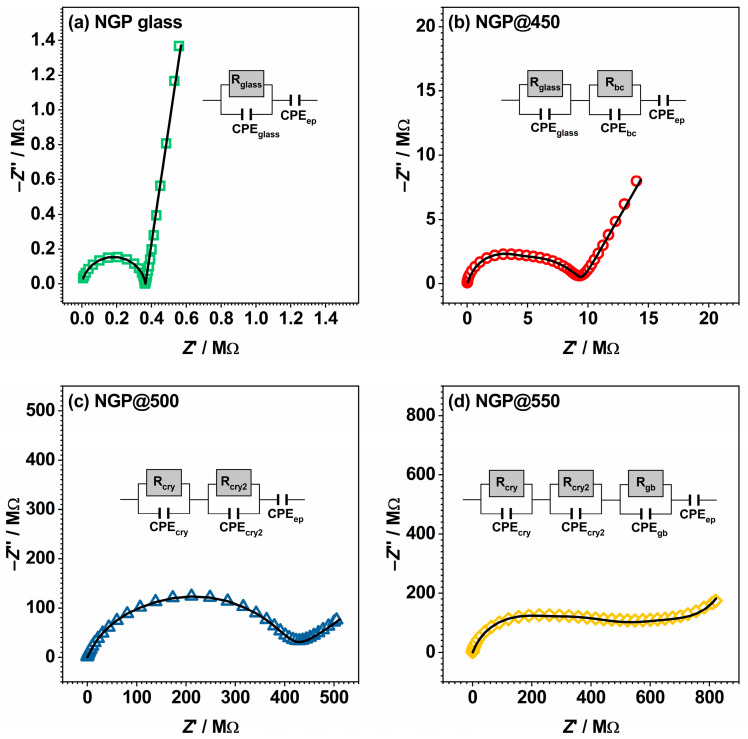
Complex impedance spectra measured at 150 °C for samples of starting glass (**a**) and prepared glass-ceramics obtained by the crystallization of starting glass at (**b**) 450 °C, (**c**) 500 °C, and (**d**) 550 °C,. The colored symbols represent experimental values, while the solid black line corresponds to the theoretical curve obtained by the fitting procedure. Each figure includes the corresponding equivalent circuit model, composed of multiple parallel combinations of the resistor (R) and the constant-phase element (CPE), used for fitting the data. The interpretation of the model is provided, with definitions as follows: glass—glassy phase, bc—beginning of crystallization, cry—crystal phase, gb—grain boundary, and ep—electrode polarization.

In order to illuminate the various contributions to total conductivity, the experimental results are presented in the form of Nyquist plots, see Figure 5. The complex impedance spectra of the investigated samples underwent substantial changes as the initial glass crystallized during the HT process, progressing from 450 °C to 550 °C. The initial glass displayed a single depressed semicircle with a noticeable EP effect, while the crystallized products revealed two or more depressed semicircles, and the influence of EP gradually waned with rising HT temperature. Since the shape of each complex impedance spectrum was directly affected by various processes and effects, such as the presence of crystal and/or amorphous phases (glass matrix), crystal grain boundaries, and EP, the distinct shapes in the complex impedance spectra resulted from the impact of HT on the microstructure of the obtained GCs. By employing modeling with the appropriate electrical equivalent circuit (EEC), the resulting fitting parameters, specifically resistance and capacitance, can be linked to each process or effect, thus assigning a physical significance to them [36]. The EECs used for modeling the samples from this study are presented in Figure 5, and the corresponding fitting parameters are listed in Table S1 in the SM.

As mentioned earlier, the spectrum of the initial NGP glass is characterized by a single high-frequency semicircle representing the bulk conductivity of the glass sample and a low-frequency linear segment, indicative of EP, which is a signature of ionic conductivity, see Figure 5a. The corresponding EEC model describing the experimental impedance spectrum comprises a parallel R-CPE circuit, where R stands for resistance, CPE represents the constant-phase element, and EP is represented through a series-connected CPE. Conversely, the complex impedance spectrum of the NGP@450 is represented by an EEC model incorporating two R-CPE circles and an additional series-connected CPE, as depicted in Figure 5b. The capacitance values of the high-frequency semicircle are on the order of ~10^−12^ F, see Table S1, indicating its association with the predominant glass matrix phase. Furthermore, the low-frequency semicircle, with a capacitance on the order of ~10^−11^ F, is correlated with the early stage of crystallization observed in this sample. The correlation between this observation and the beginning of crystallization in this sample is substantiated by the aforementioned results derived from PXRD, SEM-EDS, and IR-ATR analyses. The identical EEC model was utilized for interpreting the complex impedance spectrum of the NGP@500 sample. Nonetheless, as this sample was primarily composed of the NaPO_3_ crystalline phase and minority GeP_2_O_7_ phase, the first contribution was ascribed to the former crystalline phase, while the second contribution can be attributed to the latter one. This is further corroborated by the similar capacitance values of the two contributions, both falling within the order of magnitude of ~10^−11^ F, see Table S1.

Increasing the HT temperature to 550 °C resulted in an intriguing change in the shape of the complex impedance spectrum. To analyze the spectrum of the NGP@550 sample, a model featuring three R-CPE circuits with an additional series-connected CPE element was applied, as depicted in Figure 5d. While this sample maintained nearly the same composition, with identical crystal phases and similar relative proportions with respect to NGP@500, its impedance spectrum revealed an extra semicircle. Based on the capacitance values acquired through EEC modeling, which were approximately in the order of 10^−12^ F, 10^−11^ F, and 10^−9^ F, the first contribution was ascribed to the prevailing NaPO_3_ crystal phase, the second one was assigned to the minor GeP_2_O_7_ phase, and the third one was a result of the crystal grain boundary. The distinct contributions from the crystal grain boundary and crystalline phases in this GC sample emerged due to the formation of clearly defined crystal grains and sharp grain boundaries, see Figure 3a–c. This pronounced change in morphology profoundly influenced the electrical transport within the prepared GC samples. Aside from the observed modifications in the shape of the depressed semicircles and the number of distinct contributions due to microstructural changes, the impact of the HT temperature on the extent of electrode polarization, which is linked to sodium ion mobility [37,38], is also worth noting. It can be observed that EP gradually decreased with increasing HT temperature, indicating a reduction in sodium ion mobility within the prepared GC. This can be attributed to the promoted crystallization of the dominant NaPO_3_ crystalline phase, which led to a reduction of mobile Na^+^ ions within the remaining glass matrix. This aspect will be further discussed in the following sections.

#### 3.2.3. DC Conductivity and Activation Energy

**Figure 6:**
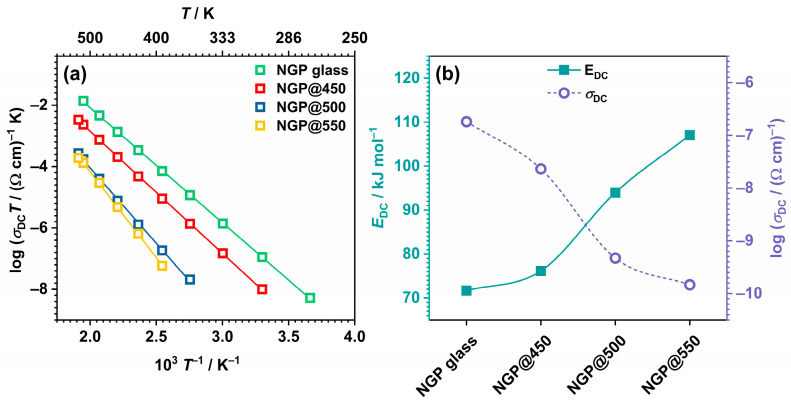
Dependence of (**a**) log(*σ*_DC_) vs. 10^3^/*T* and (**b**) *E*_DC_ and *σ*_DC_ @393 K on the crystallization temperature for all prepared samples.

From the obtained conductivity spectra, see Figure 5, the values of DC conductivity, *σ*_DC_, were determined from the DC plateau, and where it was not possible to determine them from experimental data, they were calculated from the value of the total resistance, *R*_tot_, obtained by modeling the impedance spectra in the complex impedance plane. The activation energy for DC conductivity, *E*_DC_, was calculated for all samples according to Equation (1) [37]:

*σ*_DC_*T* = *σ*_0_*exp(−*E*_DC_/k_B_*T*), (1)

where *T* is the temperature, *σ*_0_* is the pre-exponential factor, and k_B_ is Boltzmann’s constant. The dependence of *σ*_DC_ on *T* is shown in Figure 6a, while the values of *σ*_DC_ at 393 K and of *E*_DC_ are listed in Table 1, and their dependence on the crystallization temperature is shown in Figure 6b.

As the crystallization temperature increased, *σ*_DC_ continuously decreased, whereas the activation energy demonstrated the expected opposite trend. As previously explained, the observed trends are likely a consequence of the enhanced crystallization of the NaPO_3_ phase, depleting the remaining glass matrix of mobile Na^+^ ions. Nonetheless, the crystal grains were not connected enough to achieve an increase in conductivity by creating easy conduction pathways at the grain boundaries.

As the GC samples from this study predominantly consisted of the NaPO_3_ crystalline phase, the measured values of *σ*_DC_ and *E*_DC_ were compared with those of the crystalline NaPO_3_. The crystalline NaPO_3_ demonstrated an ionic conductivity of 6.1 × 10^−11^ Ω^−1^ cm^−1^ at 60 °C, with an activation energy of 0.66 eV [22]. This closely aligns with the *σ*_DC_ and *E*_DC_ values observed in the studied GC samples, where the conductivity spanned from 4.10 × 10^−10^ for NGP@450 to 1.58 × 10^−12^ Ω^−1^ cm^−1^ for NGP@550 at 60 °C, and the activation energy was within the range of 0.79–1.11 eV.

### 3.3. Dielectric Analysis

#### 3.3.1. Complex Permittivity

**Figure 7:**
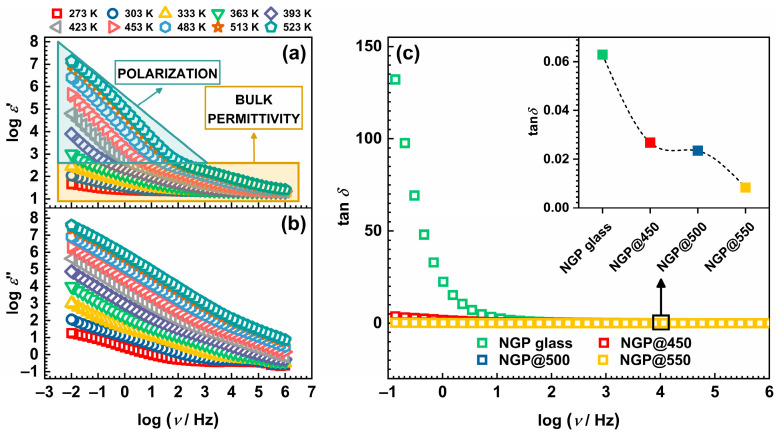
Frequency dependence of (**a**) real, *ε*′(*ω*), and (**b**) imaginary, *ε*″(*ω*), parts of the complex permittivity at different temperatures for the NGP@500 glass-ceramic, and (**c**) the loss factor, tan *δ*, for all samples @303 K. Inset: Compositional dependence of tan *δ* for all samples @303 K and 10 kHz. The lines connecting data points are a guide to the eye.

The dielectric properties of the prepared GCs were thoroughly analyzed using complex permittivity, ε*(*ω*), as defined by Equation (2) [38]:

ε*(*ω*) = 1/(*iωC*_0_Z*) = *ε*′(*ω*) – *iε*″(*ω*), (2)

where *ε*′(*ω*) and *ε*″(*ω*) are the real and imaginary parts of the complex permittivity. The real component, *ε*′(*ω*), is commonly known as the dielectric constant. The frequency dependences of *ε*′(*ω*) and *ε*″(*ω*) for the NGP@500 sample as representative of all the studied samples are shown in Figure 7a,b.

The frequency-dependent behavior of *ε*′(*ω*), which represents the dielectric permittivity, revealed two distinct characteristics stemming from polarization and bulk permittivity [37,38]. At the highest frequencies and lowest temperatures, *ε*′(*ω*) attained a constant value, as fast polarization processes took place within the sample under the applied field. As the frequency decreased, *ε*′(*ω*) exhibited a stepwise increase due to the polarization of the mobile ions regarding the fixed glass matrix. At the lowest frequencies and highest temperatures, *ε*′(*ω*) rapidly increased due to large bulk polarization, which was a consequence of charge separation at the external electrodes in contact with the sample. This phenomenon is known as electrode polarization, and it arose from the accumulation of mobile Na^+^ ions near the blocking gold electrodes that prevented the transfer of mobile ions into the external circuit [37,38]. However, here it should be noted that polarization in GC samples can also take place on a mesoscopic scale, attributed to the separation of charges at inner dielectric boundary layers known as Maxwell/Wagner/Sillars polarization [38,39]. While the primary process contributing to polarization in the samples from this study arose from the aforementioned electrode polarization, the presence of Maxwell/Wagner/Sillars polarization was also taken into account. This is because the samples in question were heterogeneous composite materials, comprising both an amorphous glass matrix and crystalline grains, which may have collectively contributed to the overall polarization. Furthermore, as the processes of polarization and conduction in ionic materials, such as the glass and GCs studied herein, were integrated into a single continuous process, the *ε*″(*ω*) curve demonstrated a linear increase with decreasing frequency. This is attributed to translational diffusion, i.e., the long-range movement of mobile ions associated with DC conductivity [37,38]. The values of *ε*′(*ω*) measured at 303 K and 10 kHz for the investigated glass and GCs can be found in Table 1. Notably, controlled crystallization of the initial glass resulted in a jump in the values of the dielectric constant, and all GC samples showed enhanced dielectric constant values around 20, with slight variations among the three GCs.

Another important parameter that can be extracted from the permittivity data is the loss factor, known as tan *δ*. This factor quantifies the phase difference resulting from energy dissipation within the sample at a specific frequency and is calculated as tan *δ* = *ε*″(*ω*)/*ε*′(*ω*) [38]. Generally, dielectric losses are notably lower at higher frequencies compared to lower frequencies and specific temperatures. This frequency-dependent behavior of tan *δ* is typically associated with conduction losses and aligns with the observations made in the glass-ceramic samples examined in this study, as illustrated in Figure 7c. The measured tan *δ* values for all samples at 303 K and 10 kHz are listed in Table 1, and the inset in Figure 7c illustrates how the loss factor varies with the HT temperature. While all samples demonstrated relatively low dielectric loss values, it was observed that the controlled crystallization of the initial glass led to a substantial reduction in tan *δ* values, decreasing from 0.063 to 0.027 for NGP@450 GC. Moreover, the subsequent increase in HT temperature additionally influenced tan *δ*, with NGP@550 GC achieving the lowest value of 0.008. Interestingly, the dependence of tan *δ* on the HT temperature closely mirrored the trend in *σ*_DC_, see Figure 6b, with the NGP@550 GC displaying the lowest values of both tan *δ* and *σ*_DC_. These findings highlight the clear impact of HT temperature on dielectric parameters, along with their correlation with *σ*_DC_, in line with prior research results [30,31]. The declining trends in both the DC conductivity and dielectric parameters observed in the three GC samples may have stemmed from multiple factors, including the increasing degree of crystallinity and morphological changes associated with elevated HT temperatures [40]. Specifically, as elucidated earlier, the increase in HT temperature resulted in the formation of larger crystalline grains, consequently leading to a reduction in their specific surface area. This, in turn, may have been affecting the polarization arising from the accumulation of space charge at grain boundary interfaces, leading to different values of dielectric parameters observed in the three GC samples. Moreover, the overall permittivity was influenced by both the intrinsic nature of the glass matrix and the crystalline phases present, with each phase contributing to the total permittivity and thereby impacting the values of the dielectric parameters.

#### 3.3.2. Electrical Modulus

Another way to observe and interpret relaxation processes is to display the results of impedance spectroscopy using the complex electric modulus, M*(*ω*). The modulus is the reciprocal of the value of the complex dielectric permittivity, ε*(*ω*), and is defined according to Equation (3) [38]:

M*(*ω*) = 1/ε*(*ω*) = *M*′(*ω*) + *iM*″(*ω*), (3)

The main advantage of using this formalism lies in reducing the effect of electrode polarization. Figure S4 in the SM shows the imaginary modulus for the starting NGP glass and GCs. A detailed analysis can also contribute to the understanding of electrical transport in GCs. The different processes present in crystallized samples are visible and easy to separate based on the value of resistance and capacity, i.e., relaxation time, using a graphic display of the frequency-dependent electrical modulus, *M*″(*ω*), and imaginary component of the impedance, *Z*″(*ω*). Both of these displays reveal one or more distinct maxima at specific frequencies, describing the corresponding relaxation process, which shifts towards higher frequencies with an increase in temperature. The frequency values of these relaxations can be correlated with the separation of the different contributions of individual phases to the total conductivity, which provides insight into the mechanisms of conductivity in the investigated GCs and the influence of crystallization on the transfer of Na^+^ ions as charge carriers.

**Figure 8:**
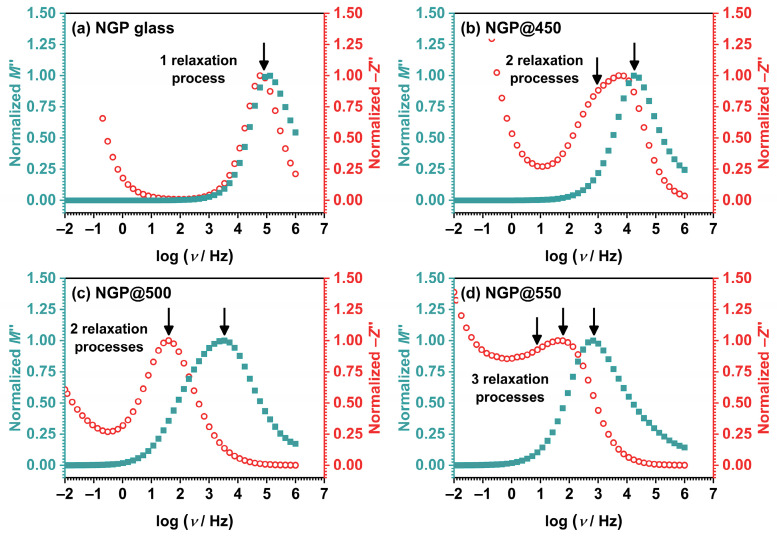
Frequency dependence of normalized *M*″(ω) and *Z*″(ω) curves for all samples at 423 K: NGP glass demonstrates a single relaxation process attributed to ionic conductivity (**a**); NGP@450 displays two relaxation processes arising from the predominant amorphous phase and an early stage of crystallization (**b**); NGP@500 exhibits two relaxation processes associated with two crystalline phases, predominantly NaPO_3_ and a minority of GeP_2_O_7_ (**c**); NGP@550 reveals three relaxation processes, stemming from the two crystalline phases, mainly NaPO_3_ and a minority of GeP_2_O_7_, as well as the grain boundary. Distinct maxima at specific frequencies, indicated by black arrows, correspond to one or more different relaxation processes observed in glassy and crystallized samples.

For an investigation into whether the observed peaks in the frequency-dependent *M*″(*ω*) and *Z*″(*ω*) curves correspond to the same relaxation process, the curves were normalized, as demonstrated in Figure 8.

It was indeed observed that for the initial NGP glass, as can be seen in Figure 8a, the maxima in both the *M*″(*ω*) and *Z*″(*ω*) curves were closely aligned, however do not completely overlap, suggesting the distribution of relaxation time, and presence of components from both long-range and localized relaxation. This behavior suggests that the observed phenomenon was a manifestation of the same underlying process, i.e., ionic conductivity [37,38]. In contrast to the initial glass, the GC samples, which constituted heterogeneous systems comprising uniformly distributed crystalline grains within a glassy matrix, exhibited distinct characteristics. This is evident in the shape of the *Z*″(*ω*) curve for the NGP@450 sample, which displayed two overlapped maxima. In contrast, a single maximum was observed in the *M*″(*ω*) curve, and it closely matches the second of the two maxima in the *Z*″(*ω*) curve, see Figure 8b. Since *M*″(*ω*) reflects processes associated with reduced capacitance values, the presence of just one peak in the *M*″(*ω*) curve implies that the prevailing process at higher frequencies was characterized by diminished capacitance values when compared to the process at lower frequencies. Given the substantial weight fraction of the remaining glassy matrix in NGP@450 GC, the high-frequency contribution is ascribed to the predominant amorphous phase, while the lower-frequency process might be associated with an early stage of crystallization in this sample. This is further corroborated by the EEC modeling, see Figure 5b. Furthermore, the frequency dependence of *M*″(*ω*) for the NGP@500 sample reveals contributions from two separate relaxation processes, which overlap due to similar relaxation frequencies. Conversely, a single peak was observed in the *Z*″(*ω*) curve, closely aligning with the first of the two maxima in the *M*″(*ω*) curve, as depicted in Figure 8c. Considering that *Z*″(*ω*) reflects processes characterized by higher resistance values, the presence of only one maximum in the *Z*″(*ω*) curve suggests that the process occurring at a lower frequency exhibited higher resistance values compared to the process observed at a higher frequency, effectively masking the latter. Taking into account weight fractions of two crystalline phases in NGP@500 GC, the high-frequency contribution is attributed to the majority NaPO_3_ crystalline phase, while the lower-frequency process may be linked to the minority GeP_2_O_7_ crystalline phase, as previously depicted in Figure 5c. In the case of NGP@550, both the *M*″(*ω*) and *Z*″(*ω*) curves exhibit distinct individual contributions, much like those observed for NGP@500, see Figure 8d. Interestingly, despite NGP@550 GC having nearly equal weight fractions of two crystalline phases, NaPO_3_ and GeP_2_O_7_, with respect to the NGP@500 GC, their *M*″(*ω*) and *Z*″(*ω*) curves exhibit differing shapes, a phenomenon also observed in their complex impedance planes, see Figure 5c,d. Namely, the *Z*″(*ω*) curve of NGP@550 GC is broader compared to the *Z*″(*ω*) curve of NGP@500 GC, which can be explained by the third contribution observed at the lowest frequencies. However, the three relaxation maxima overlap, making it challenging to separate them. On the other hand, in the *M*″(*ω*) curve, the most prominent contribution is at the highest frequencies, and this can be attributed to the majority NaPO_3_ phase (74.3%). Therefore, the processes characterized by the highest resistance appear to be a result of the presence of the additional GeP_2_O_7_ crystal phase and contributions from the grain boundary. The distinct contributions of the crystal grain boundary and the crystalline phases in NGP@550 GC emerged due to the formation of well-defined crystal grains and sharp grain boundaries. An elevated HT temperature may also have contributed to the sintering of crystal grains and reduced their specific surface area. As a result, the morphology of NGP@550 GC appears significantly more compact, as shown in Figure 3, in contrast to NGP@500 GC, whose microstructure exhibits a more porous structure, as depicted in Figure 2c,d.

## 4. Conclusions

This study explored the (micro)structural, electrical, and dielectric properties of glass-ceramics produced through a 24 h heat-treatment (HT) of initial glass with a composition of 40Na_2_O-10GeO_2_-50P_2_O_5_ at three different temperatures (450, 500, and 550 °C). The results obtained from PXRD, IR-ATR, and SEM-EDS analyses demonstrate a progressive increase in crystallinity with rising HT temperature. NaPO_3_ emerged as the primary crystalline phase in all samples, while the GeP_2_O_7_ crystalline phase was present in the minority. SEM-EDS findings reveal that the @450 °C sample comprised an amorphous glass matrix with nanometric crystalline grains, while the @500 °C sample exhibited randomly scattered crystalline grains lacking distinct morphology. Despite having a composition similar to the previous two samples, the @550 °C sample displayed larger micron-scale NaPO_3_ crystalline grains with a square-shaped morphology. The results of impedance spectroscopy revealed a strong correlation between electrical and dielectric properties and controlled crystallization conditions. The electrical equivalent circuit modeling of complex impedance spectra, coupled with complex electrical modulus and impedance analyses, evidenced contributions from the glass matrix, grain boundaries, and/or crystalline phases. Comparing the GCs to the initial glass, a decrease in DC conductivity was observed with an increase in HT temperature, attributed to the crystallization of the NaPO_3_ phase depleting the glass matrix of mobile Na^+^ ions. On the other hand, the dielectric parameters of the prepared GCs were improved, with a substantial increase in dielectric constant values (~20) and a simultaneous decline in dielectric loss values (~10^−3^) as HT temperatures rose. Such effects may be related to both the intrinsic nature of the glass matrix and the crystalline phases present, with each phase contributing to the permittivity and thereby impacting the values of the dielectric parameters. Particularly, the GC obtained at 450 °C stood out with its highest dielectric constant and low dielectric loss, along with moderate ionic conductivity. Overall, this research provides valuable insights and uncovers the relationship between the heat-treatment conditions, microstructure, and functional properties of prepared glass-ceramics, which highlights the importance of controlled crystallization for fine-tuning material properties tailored for potential applications paving the way for future developments in advanced materials science.

## Figures and Tables

**Table 1:** DC conductivity, *σ*_DC_, activation energy for DC conductivity, *E*_DC_, and selected dielectric properties for all studied samples.

Sample	*σ*_DC_ (Ω^−1^ cm^−1^) ^a^	*E*_DC_ (kJ mol^−1^)	*ε*′(*ω*) ^b^	tan*δ* ^b^
NGP glass	1.81 × 10^−7^	71.7	13.24	0.063
NGP@450	2.30 × 10^−8^	76.1	22.82	0.027
NGP@500	4.70 × 10^−10^	94.0	20.98	0.023
NGP@550	1.47 × 10^−10^	107.0	19.37	0.008

^a^ at 393 K; ^b^ at 303 K and 10 kHz.

## Data Availability

The data presented in this study are available from the corresponding author upon request.

## Supplementary Materials

The following supporting information can be downloaded at: https://www.mdpi.com/article/10.3390/ma17020306/s1, Figure S1: (a) IR-ATR spectra of all samples from this study and (b) comparison of IR-ATR apectra of NGP@500 GC and NaPO_3_; Figure S2: SEM images and EDS spectra of the NGP@450 glass-ceramic surface: (a) area, (b) surface—separation and (c) surface—separation 2; Figure S3. SEM images and EDS spectra of the NGP@500 glass-ceramic surface; Figure S4: Spectra of imaginary moduli, *M*′′(ω), for all samples. Table S1: Fitting parameters obtained from EEC modeling of complex impedance spectra measured at 150 °C for the initial glass and glass-ceramics from this study.
