# Supplementary material for: The Crystallization Behavior of a Na2O-GeO2-P2O5 Glass System: A (Micro)Structural, Electrical, and Dielectric Study
Source: Materials (Basel). 2024 Jan 7;17(2):306. doi: 10.3390/ma17020306 (PMC10817665; doi:10.3390/ma17020306)
Supplement: Supplementary file 1 [file materials-17-00306-s001.zip › materials-2787554-Supplementary.pdf]

materials

MDPI

Supplementary Materials

The Crystallization Behavior of a Na_2O-GeO_2-P_2O_5 Glass System: A (Micro)Structural, Electrical, and Dielectric Study

Sara Marijan [1], Marta Razum [1], Kristina Sklepić Kerhač [1], Petr Mošner [2], Ladislav Koudelka [2], Jana Pisk [3], Andrea Moguš-Milanković [1], Željko Skoko [4,*] and Luka Pavić [1,*]

[1] Division of Materials Chemistry, Ruđer Bošković Institute, Bijenička 54, 10000 Zagreb, Croatia; smarijan@irb.hr (S.M.); mrazum@irb.hr (M.R.); ksklepickerhac@gmail.com (K.S.K.); mogus@irb.hr (A.M.-M.)
[2] Department of General and Inorganic Chemistry, Faculty of Chemical Technology, University of Pardubice, 53210 Pardubice, Czech Republic; petr.mosner@upce.cz (P.M.); ladislav.koudelka@upce.cz (L.K.)
[3] Department of Chemistry, Faculty of Science, University of Zagreb, Horvatovac 102a, 10000 Zagreb, Croatia; jana.pisk@chem.pmf.hr
[4] Department of Physics, Faculty of Science, University of Zagreb, Bijenička 32, 10000 Zagreb, Croatia
* Correspondence: zskoko@phy.hr (Ž.S.); lpavic@irb.hr (L.P.)

Citation: Marijan, S.; Razum, M.; Sklepić Kerhač, K.; Mošner, P.; Koudelka, L.; Pisk, J.; Moguš-Milanković, A.; Skoko, Ž.; Pavić, L. Crystallization Behavior of Na_2O-GeO_2-P_2O_5 Glass System: (Micro)Structural, Electrical, and Dielectric Study. *Materials* **2024**, *17*, 306. https://doi.org/10.3390/ma17020306

Academic Editor: Pengfei Wang

Received: 7 December 2023
Revised: 2 January 2024
Accepted: 5 January 2024
Published: 7 January 2024

Copyright: © 2024 by the authors. Licensee MDPI, Basel, Switzerland. This article is an open access article distributed under the terms and conditions of the Creative Commons Attribution (CC BY) license (https://creativecommons.org/licenses/by/4.0/).

1. IR-ATR spectroscopy

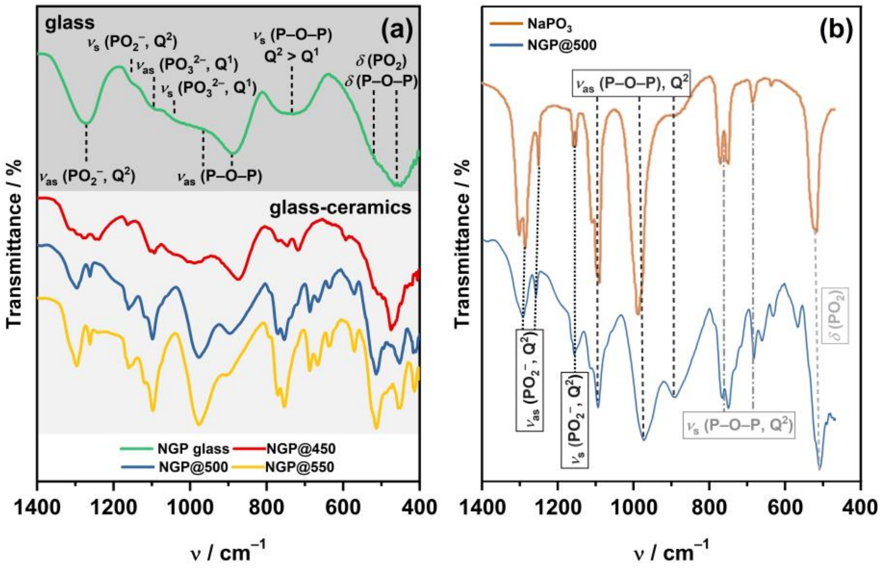

Figure S1. (a) IR-ATR spectra of all samples from this study and (b) comparison of IR-ATR apectra of **NGP@500** GC and $NaPO_3$.

The IR-ATR spectrum of NGP glass, see Figure S1(a), exhibits distinct bands characteristic of metataphosphate (Q^2) glasses, and it also reveals indications of pyrophosphate units (Q^1), which is consistent with the O/P ratio of 3.1 [1]. The intense band observed at 1275 cm^{-1}, followed by shoulder at 1110 cm^{-1} is indicative of the asymmetric and symmetric stretching of non-bridging oxygen atoms in Q^2 units, respectively [2,3], while the bands at 970 cm^{-1} and 890 cm^{-1} can be attributed to the asymmetric stretching vibrations of P–O–P groups in metaphosphate units, manifesting as chains, rings, and terminal groups [2,3]. Additionally, low intensity shoulders at 1150 cm^{-1} and 1035 cm^{-1} can be assigned to asymmetric and symmetric stretching of non-

bridging oxygen atoms in Q^1 units, respectively, which presence in small quantities is in accordance with O/P ratio in studied glass. Furthermore, the symmetric stretching vibrations of P–O–P within Q^2 and Q^1 units are found in the range between 640–810 cm^{-1} [2,3]. Moreover, the bands around ~460 cm^{-1} and ~520 cm^{-1} can be ascribed to the bending vibrations of P–O–P and PO_2 units within the metaphosphate framework[2,3]. Notably, the IR-ATR spectroscopy results for the initial glass correlate well with prior Raman spectroscopy findings[1].

In contrast to the broad and diffuse bands observed in the spectrum of the initial glass, the spectra of the prepared glass-ceramics exhibit well-defined, sharp signals characteristic of crystalline materials. As the heat-treatment temperature is elevated from 450°C to 550°C, a notable transformation in the appearance of the spectra becomes apparent. The spectrum of NGP@450 glass-ceramic exhibits initial signs of crystallization and the presence of a glass matrix. In contrast, the spectra of NGP@500 and NGP@550 glass-ceramics display remarkable similarity, reflecting a notably advanced stage of crystallization. Here, it's noteworthy to highlight that the acquired IR-ATR spectra of GC samples, characterized by a substantial amount of $NaPO_3$ crystal phase, exhibit a striking similarity to the results of the structural analysis of $NaPO_3$ documented in [4]. Additionally, the strong agreement observed between the IR-ATR spectra of the prepared GCs and the IR spectrum of the $NaPO_3$ crystal phase (SpectraBase®), see Figure S1(b), provides additional confirmation that $NaPO_3$ is the predominant phase within the samples.

2. SEM-EDS analysis

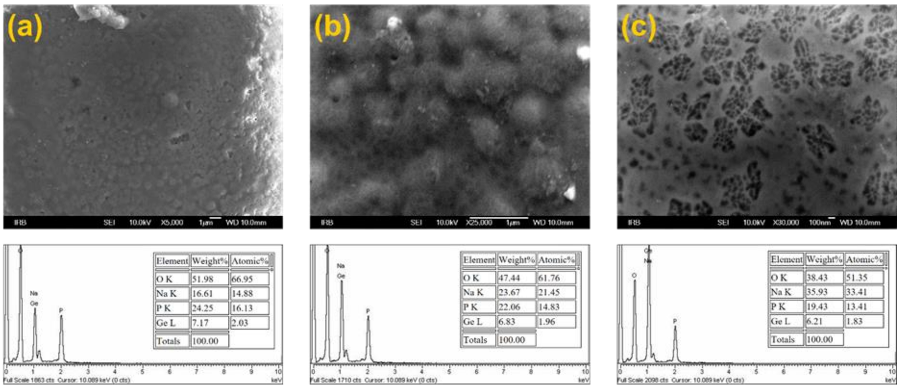

Figure S2. SEM images and EDS spectra of the **NGP@450** glass-ceramic surface: (a) area, (b) surface – separation and (c) surface - separation 2.

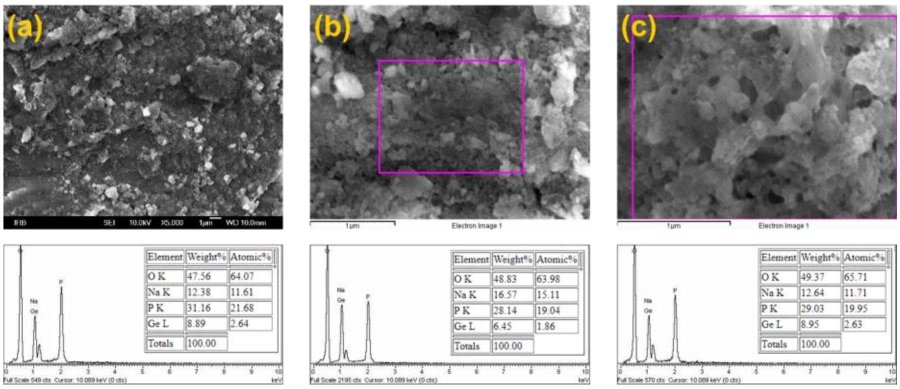

Figure S3. SEM images and EDS spectra of the NGP@500 glass-ceramic surface.

3. Electrical analysis

3.1. Complex impedance plane– electrical equivalent circuit modelling

Table S1. Fitting parameters obtained from EEC modeling of complex impedance spectra measured at 150 °C for the initial glass and glass-ceramics from this study

Parameters	NGP	NGP@450	NGP@500	NGP@550
R_1[a] (Ω)	3.65×10^{5}	3.06×10^{6}	5.03×10^{7}	1.96×10^{8}
A_1[b] (s$^{\alpha}$ Ω^{-1})	2.27×10^{-11}	9.41×10^{-12}	4.29×10^{-11}	1.42×10^{-11}
α_1[c]	0.90	0.94	0.86	0.84
C_1[d] (F)	6.22×10^{-12}	4.92×10^{-12}	1.50×10^{-11}	4.63×10^{-12}
R_2[a] (Ω)	-	6.51×10^{6}	3.59×10^{8}	2.58×10^{8}
A_2[b] (s$^{\alpha}$ Ω^{-1})	-	9.23×10^{-10}	6.56×10^{-11}	2.09×10^{-10}
α_2[c]	-	0.60	0.71	0.66
C_2[d] (F)	-	2.95×10^{-11}	1.38×10^{-11}	4.80×10^{-11}
R_3[a] (Ω)	-	-	-	4.12×10^{8}
A_3[b] (s$^{\alpha}$ Ω^{-1})	-	-	-	2.77×10^{-9}
α_3[c]	-	-	-	0.49
C_3[d] (F)	-	-	-	3.18×10^{-9}
A_4[b] (s$^{\alpha}$ Ω^{-1})	8.85×10^{-6}	2.81×10^{-7}	2.37×10^{-8}	7.16×10^{-8}
α_4[c]	0.91	0.67	0.40	0.81

[a] Individual resistance (R) values for each R-CPE circuit element in the proposed model. [b] A – a constant (CPE capacitance) derived from the empirical impedance function, $Z_{CPE}^{*} = 1/A(i\omega)^{\alpha}$, measured in s$^{\alpha}$ Ω^{-1} unit. [c] α – a constant ($0 \le \alpha \le 1$) determined from the empirical function, $Z_{CPE}^{*} = 1/A(i\omega)^{\alpha}$. When $\alpha = 1$ (true capacitor), the unit becomes s Ω^{-1} = F. [d] Individual capacitance (C) values calculated for each R-CPE circuit element in the proposed model using the formula $C = A(\omega_{max})^{\alpha-1}$.

4. Dielectric analysis

4.1. Complex Modulus Formalism

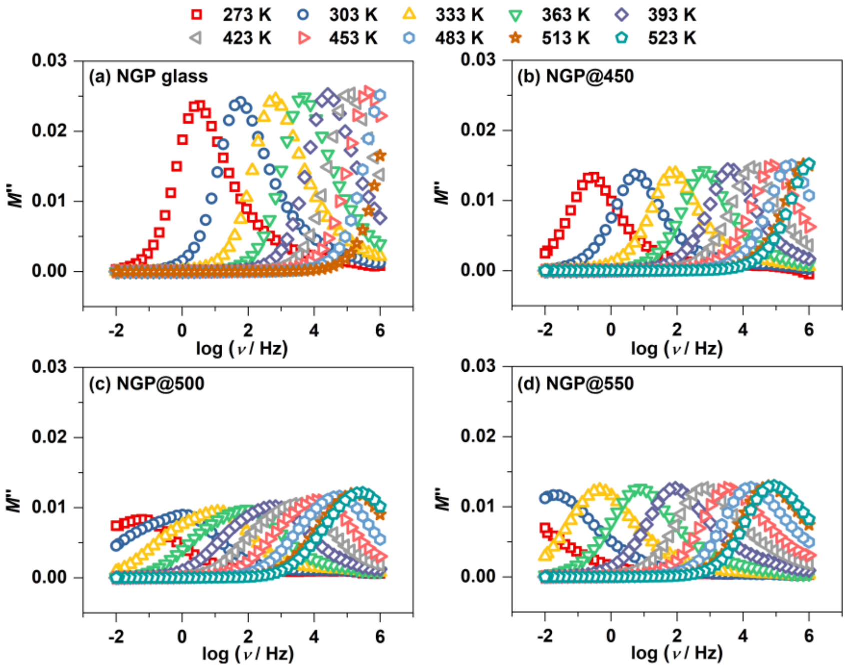

Figure S4. Spectra of imaginary moduli, $M''(\omega)$, for all samples.

References

1. Sklepić, K.; Tricot, G.; Mošner, P.; Koudelka, L.; Moguš-Milanković, A. Sodium Ion Conductivity in Mixed Former Na_2O–P_2O_5–GeO_2 and Na_2O–B_2O_3–P_2O_5–GeO_2 Glasses. *J. Phys. Chem. C* **2021**, *125*, 10593–10604, doi:10.1021/acs.jpcc.1c00072.
2. Moustafa, Y.M.; El-Egili, K. Infrared Spectra of Sodium Phosphate Glasses. *J. Non-Cryst. Solids* **1998**, *240*, 144–153, doi:10.1016/S0022-3093(98)00711-X.
3. Sahar, M.R.; Hussein, A.W.M.A.; Hussin, R. Structural Characteristic of Na_2O-P_2O_5-GeO_2 Glass Systems. *J. Non-Cryst. Solids* **2007**, *353*, 1134–1140, doi:10.1016/j.jnoncrysol.2006.12.032.
4. Trivedi, S.; Pamidi, V.; Fichtner, M.; Reddy, M.A. Ionically Conducting Inorganic Binders: A Paradigm Shift in Electrochemical Energy Storage. *Green Chem.* **2022**, *24*, 5620–5631, doi:10.1039/D2GC01389D.
